# Apatinib Plus Temozolomide: An Effective Salvage Treatment for Recurrent Glioblastoma

**DOI:** 10.3389/fonc.2020.601175

**Published:** 2021-02-04

**Authors:** Jingjing Ge, Cheng Li, Fengjun Xue, Shaopei Qi, Zhimeng Gao, Chunjiang Yu, Junping Zhang

**Affiliations:** ^1^ Department of Neuro-Oncology, Sanbo Brain Hospital, Capital Medical University, Beijing, China; ^2^ Department of Neurosurgery, Sanbo Brain Hospital, Capital Medical University, Beijing, China

**Keywords:** glioblastoma, apatinib, angiogenesis inhibitors, temozolomide, VEGFR-2, chemotherapy

## Abstract

**Background:**

Treatment for recurrent glioblastoma is poor, and there is a need for better therapies. Here we retrospectively assessed the efficacy and toxicity of temozolomide plus apatinib, an oral small-molecule tyrosine kinase inhibitor targeting vascular endothelial growth factor receptor 2 in recurrent glioblastoma.

**Materials and Methods:**

A retrospective analysis of patients with recurrent glioblastoma who underwent apatinib plus temozolomide treatment was performed. Apatinib was given at 500 mg once daily. Temozolomide was administered at 200 mg/m^2^/d on days 1–5 or 50 mg/m^2^/d continuous daily according to whether they had experienced temozolomide maintenance treatment before. The main clinical data collected included tumor characteristics, status of MGMT promoter, and IDH mutation, number of relapse, response, survival, adverse reactions, and salvage therapies.

**Results:**

From April 2016 to August 2019, thirty-one patients were identified. The objective response rate was 26.3%, and the disease control rate was 84.2%. The progression-free survival (PFS) at 6 months and overall survival (OS) at 12 months were 44.6 and 30.2%. The median PFS and OS were 4.9 and 8.2 months, respectively. Two patients achieved long PFS of 30.9 and 38.7+ months. The median survival time after progression of the patients with or without salvage bevacizumab was 5.1 *versus* 1.2 months. The most common grade 3 or 4 toxicities were hypertension (5.8%), decreased appetite (5.8%), and thrombocytopenia (4.3%), most of which were resolved after symptomatic treatment or dose reduction.

**Conclusion:**

Apatinib plus temozolomide is an effective salvage regimen with manageable toxicities for recurrent glioblastoma and could not reduce the sensitivity to bevacizumab.

## Introduction

Glioblastoma is the most common and aggressive group of primary central nervous system tumors (1). In 2005, Stupp and colleagues established a standard treatment for newly diagnosed glioblastoma consisting of concomitant chemoradiotherapy with temozolomide and then maintenance treatment with temozolomide for 6 to 12 months. However, even with current standard treatment, the median overall survival (OS) and progression-free survival (PFS) are only 14.6 and 6.9 months respectively for glioblastoma (2). Tumors ultimately recur, killing most of patients.

To our knowledge, no standard of care has been established for patients with recurrent glioblastoma. Besides histological hallmarks of anaplasia, cell proliferation, and necrosis, glioblastoma is characterized by marked microvascular proliferation and vascular permeability (3). Thus, anti-angiogenesis is a promising therapeutic strategy and vascular endothelial growth factor (VEGF) signaling is one of the best-characterized key therapeutic targets.

The most extensively investigated regimen for recurrent glioblastoma is bevacizumab, a humanized monoclonal antibody that inhibits VEGF. Based on two prospective Phase II trials with non-inferior response rate and PFS rate at 6 months (6m-PFS) of 36%, bevacizumab was approved by the US Food and Drug Administration for recurrent glioblastoma in 2009 (4, 5). In the EORTC 26101 phase 3 trial, for the glioblastoma with a first progression after standard chemoradiotherapy, added bevacizumab prolonged the median PFS, which was 4.2 months for the bevacizumab plus lomustine group and 1.5 months for the lomustine group (HR 0.49). The 6m-PFS was 30.2% for bevacizumab plus lomustine group and 16.9% for the lomustine group. However, the combined therapy did not confer a survival advantage over the monotherapy: 9.1 months *versus* 8.6 months (6). Moreover, there are no effective salvage therapies after bevacizumab failure (7).

In the VEGF signaling family, vascular endothelial growth factor receptor-2 (VEGFR-2) is the primary receptor that mediates angiogenesis (8). Apatinib, a small-molecule tyrosine kinase inhibitor that is administered orally, selectively binds to and strongly inhibits VEGFR-2. Apatinib was approved to treat patients with advanced gastric cancer refractory to two or more lines of prior chemotherapy in China (9). Several studies have revealed that apatinib also significantly improved the survival of intermediate and advanced hepatocellular carcinoma (10), ovarian cancer (11), and advanced non-squamous non-small cell lung cancer (12). In addition, apatinib can reverse multidrug resistance and enhance the efficacy of some conventional anticancer drugs, such as doxorubicin, vincristine, and verapamil (13, 14). In a preclinical study, apatinib suppressed glioma cell growth and metastasis and promoted anti-tumor activity of temozolomide (15).

In this study, we investigated the efficacy and safety of apatinib in combination with temozolomide in patients with recurrent glioblastoma.

## Materials and Methods

### Patients

We performed a retrospective study for all the patients with recurrent glioblastoma treated with apatinib plus temozolomide in Sanbo Brain Hospital, Capital Medical University between April 2016 and August 2019. This study was approved by the Ethics Committee of Sanbo Brain Hospital, Capital Medical University.

Inclusion criteria were as follows: Age at 18–70 years old; Karnofsky Performance Score (KPS) ≥50%; histological diagnosis of glioblastoma; recurrence was confirmed histologically or by radiographic evidence; no previous treatment with bevacizumab or other antiangiogenic drugs; treated with at least one cycle of the combined regimen of apatinib and temozolomide; had at least one post-treatment radiographic follow-up. All of the patients were required to have normal hematologic, hepatic and renal function to be eligible for treatment. Patients previously use enzyme-inducing antiepileptic drugs (EIAEDs) had been switched to non-EIAEDs at least 2 weeks before treatment. Patients with incomplete medical records were excluded.

### Treatment

All patients received oral apatinib 500 mg once daily in combination with temozolomide. Temozolomide was administered at 200 mg/m^2^/d according to the standard 5/28 days regimen for patients who had not previously received temozolomide. Patients who experienced a relapse following the standard 5/28 temozolomide schedule received continuous daily temozolomide (50 mg/m^2^/d). One treatment cycle was defined as 28 days (4 weeks). Patients continued treatment until they experienced disease progression or unacceptable toxicity. Patients provided informed consent authorizing the use of their personal information for research purposes before treatment.

The clinical data collected included the following: age, sex, KPS, previous therapies, tumor size, number of relapse, status of O6-methylguanine DNA-methyltransferase (MGMT) promoter and isocitrate dehydrogenase (IDH) mutation, treatment cycles, response, dose changes, adverse reactions, progression date, salvage therapies after progression, and dead date.

### Assessments

The radiographic responses were classified according to the RANO criteria (16). Contrast-enhanced MRI was performed at baseline and every two cycles thereafter until disease progression. If complete response (CR) or partial response (PR) was achieved, MRI was conducted after the following cycle to confirm the efficacy. Diffusion and perfusion-weighted imagings were introduced to differentiate pseudoprogression from true progression. Toxicities were classified according to the Common Terminology Criteria for Adverse Events (CTCAE) 4.0.

### Statistical Analyses

The endpoints include a 6m-PFS, objective response rate, median PFS, median OS, OS rate at 12 months (12m-OS) and toxicity. PFS was defined as the time interval from treatment initiation to disease progression, death from any cause, or last follow-up, whichever occurs first. OS was defined as the time interval from treatment initiation to death from any cause or last follow-up.

Statistical analyses were performed using SPSS 24.0 software. Categorical variables were described by numbers and percentages, and continuous variables were described by median and range. Survival curves for PFS and OS were analyzed using the Kaplan–Meier method. The log-rank test was used to evaluate relations between survival and categorical predictor variables (MGMT status, IDH status, number of relapse, tumor dissemination, bevacizumab use after progression). *P* values of < 0.05 were considered statistically significant.

## Results

### Patient and Disease Characteristics

Between April 2016 and August 2019, a total of 31 patients were identified (**Table 1**). Twenty of the 31 patients (64.5%) were men, and the median age was 53 years old (range, 21–70 years). Nine patients (29%) had experienced two or more progressions before treatment. Seven patients just received radiotherapy concomitant with temozolomide, without maintenance treatment. Twenty-three patients (74.2%) experienced disease progression while on 5-day temozolomide maintenance therapy, and one got progressed 35.5 months after temozolomide discontinuation. The median time interval of last recurrence and temozolomide discontinuation was 0.9 months (range, 0–35.5 months). Seventeen (54.8%) patients had tumors with intracranial dissemination. Nineteen (61.3%) patients had tumors with the maximal diameter larger than 30 mm. Five patients received tumor total resection at the last relapse and had non-measurable lesion. Nineteen (61.3%) patients had unmethylated MGMT promoter. Twenty (64.5%) patients have IDH wild-type tumors.

**Table 1:** Baseline characteristics of patient and tumor.

Characteristic		rGBM, n = 31
Age, years		
Mean		51.5
Median		53
Range		21**–**70
Sex, n(%)		
Male		20 (64.5)
Female		11 (35.5)
Initial KPS, n(%)		
90**–**100		10 (32.3)
70**–**80		12 (38.7)
50**–**60		9 (29.0)
No. of relapse, n(%)		
First		22 (71.0)
Second		6 (19.4)
Third		3 (9.7)
No. of previous surgery, n(%)		
1		17 (54.8)
2		9 (29.0)
3		5 (16.1)
Resection at last relapse, n(%)		
Yes		9 (29.0)
No		22 (71.0)
Maximal diameter^a^, n(%)		
>30 mm		19 (61.3)
≤30 mm		7 (22.6)
non-measurable lesion		5 (16.1)
Tumor dissemination, n(%)		
No		9 (29.0)
intracranial dissemination		17 (54.8)
non-measurable lesion		5 (16.1)
Previous chemotherapy, n(%)		
TMZ, concomitant		29 (93.5)
TMZ, maintenance none		7 (22.6)
TMZ, maintenance, 5/28		24 (77.4)
Progression on TMZ, n(%)		23 (74.2)
Progression after TMZ, n(%)		1 (3.2)
Patient characteristics	rGBM, n = 31	
Median time of last recurrence [range], mo		
From diagnosis	10.8 [2.3**–**43.8]	
From TMZ discontinuation	0.9 [0**–**35.5]	
MGMT status, n(%)		
Unmethylated	19 (61.3)	
Methylated	5 (16.1)	
Not done/unknown	7 (22.6)	
IDH1/2 mutation, n(%)		
No	20 (64.5)	
Yes	2 (6.5)	
Not done/unknown	9 (29.0)	

rGBM, recurrent glioblastoma; KPS, Karnofsky performance status; TMZ, temozolomide; MGMT, O6-methylguanine DNA-methyltransferase; IDH, isocitrate dehydrogenase.

a Maximal tumor diameter determined on contrast enhanced axial, sagittal, or coronal Magnetic Resonance Imaging.

### Response to Treatment

**Figure 1:**
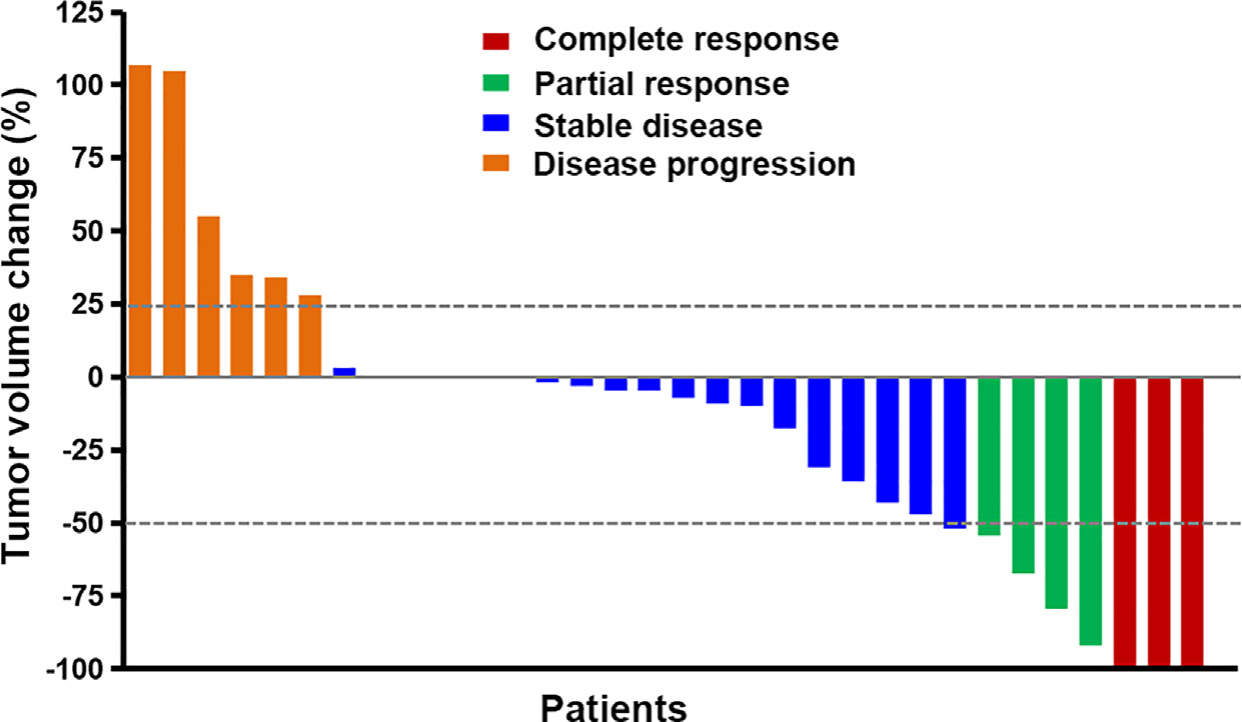
The best response to apatinib plus temozolomide treatment. Five patients had non-measurable lesion and had 0% change from baseline. The different colors indicate different responses. The response was classified according to the RANO criteria.

**Figure 2:**
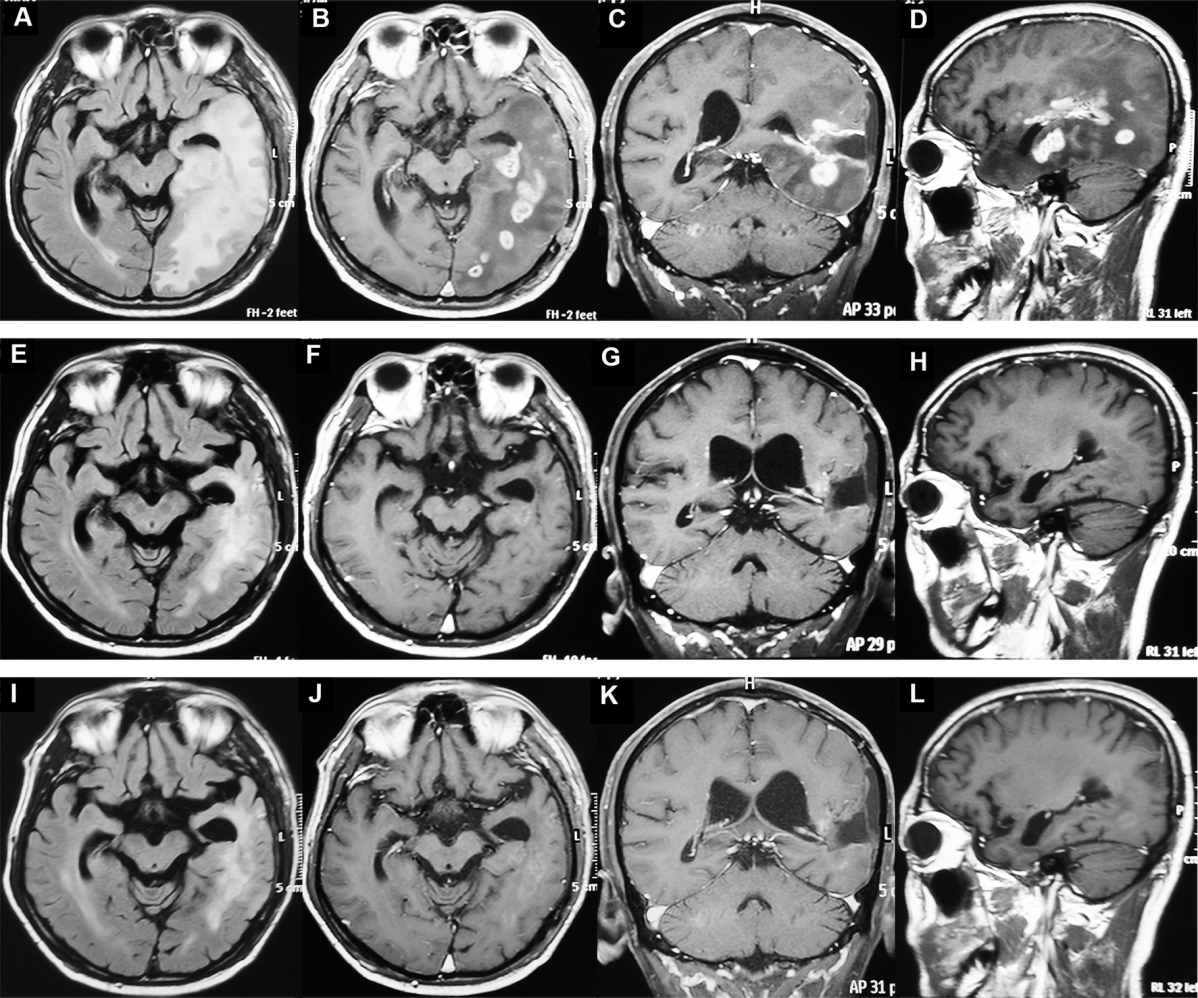
MRI of one patient with recurrent glioblastoma before and after apatinib plus temozolomide treatment. **(A–D)** The MRI before treatment. The tumors were enhanced and intracranial disseminated. **(E–H)** The MRI after two cycles of treatment. The enhanced tumors disappeared and the non-enhanced lesions (Flair) were decreased. **(I–L)** MRI after three cycles of treatment. The patient had a complete response.

The response to this combined treatment is shown in **Figure 1**. Of the 31 patients, three got CR, five got PR, 17 remained stable disease (SD) and six got progressed disease (PD). All of the five patients with non-measurable lesion achieved SD. The objective response rate (ORR) was 26.3%, and the disease control rate (DCR) was 84.2%. **Figure 2** shows the MRI changes of one patient who got CR. After receiving two cycles of therapy, the extensive enhanced tumors were obviously diminished, and the non-enhanced lesions (Flair) were decreased (**Figures 2A–H**). The response was confirmed by MRI attained after three cycles of therapy (**Figures 2I–L**).

Of the 22 patients with first relapsed glioblastoma, three got CR, four got PR, 11 got SD, and four got PD. The ORR was 31.8%, and the DCR was 81.8%.

### Efficacy

The median follow-up period was 7.9 (range, 3.2 to 38.7) months. At the last follow-up (December 31, 2019), one patient was still under treatment and one was lost to follow-up after three evaluations.

**Figure 3:**
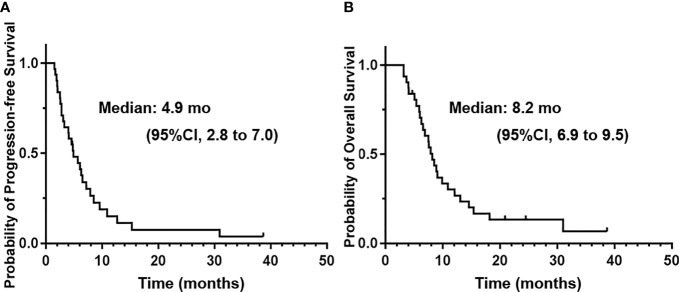
The Kaplan–Meier curves of progression-free survival **(A)** and overall survival **(B)** of the whole patients treated with apatinib plus temozolomide.

For all of the patients, the estimated 6m-PFS rate was 44.6% (95% CI, 27 to 62.2%). The 12m-OS rate was 30.2% (95% CI, 13.7 to 46.7%). The median PFS was 4.9 months (95%CI, 2.8 to 7 months). The median OS was 8.2 months (95%CI, 6.9 to 9.5 months). The median time interval between disease progressions to death was 3.2 months. **Figure 3** shows the Kaplan–Meier curves of PFS and OS of the whole population.

An exciting finding was that the two patients who discontinued treatment without tumor progression after 2 years achieved long PFS of 30.9 and 38.7+ months, while their prior recurrence intervals were only 12 and 14 months, respectively. Both of them harbored unmethylated MGMT and wild-type IDH. One received tumor total resection at the last relapse, and the other one had a local tumor without resection.

For the 20 patients with wild-type IDH, the median PFS and 6m-PFS were 5.7 months (95%CI, 3.6 to 7.8 months) and 48.7%, and the median OS and 12m-OS were 8.2 (95%CI, 6.8 to 9.6 months) and 37.1%. For the 24 patients who experienced disease progression while on 5-day temozolomide, median PFS was 5.7 months (95%CI, 2.2 to 9.2 months) and OS was 8.5 months (95%CI, 6.2 to 10.8 months). The 6m-PFS and 12m-OS were 49.4 and 35.1%, respectively.

We also analyzed the relationship between efficacy and MGMT status, number of relapse, tumor dissemination, tumor size, and bevacizumab use after progression (**Table 2**). The patients without tumor dissemination might have a better prognosis than those with dissemination (7.9 *versus* 4 months for PFS, 12.1 *versus* 7.5 months for OS). However, there was no statistical difference. After progression, nine out of 28 patients received salvage bevacizumab treatment. The median survival time after progression of the patients with or without bevacizumab was 5.1 (95%CI, 4.5 to 5.8) *versus* 1.2 (95%CI, 0 to 2.6) months. The Log-Rank *P* = 0.174, while the Breslow *P* = 0.031. This indicated that bevacizumab usage after progression may further prolong the survival time. Moreover, the efficacy might be not related to the MGMT status, number of relapse or maximal diameter of tumor, which may be due to the small sample size and need to be confirmed in a large-scale study.

**Table 2:** Survival time according to potential prognostic factors.

Prognostic Factor	No.	mPFS (months)^a^	6m-PFS(%)^b^	*P*-value^c^	mOS(months)^a^	12m-OS(%)^b^	*P*-value^c^
MGMT status							
Unmethylated	19	6.1 (4–8.2)	52.6 ± 11.5	0.791	8.5 (6.7–10.3)	36.8 ± 11.1	0.402
Methylated	5	10.9^d^	60 ± 21.9		9.9 (5–14.8)	26.7 ± 22.6	
Number of relapse							
1	22	4.9 (3.3–6.5)	44.3 ± 10.8	0.905	8.2 (6.7–9.7)	33.6 ± 10.3	0.997
>1	9	4 (2–6)	44.4 ± 16.6		7.9 (5–10.8)	22.2 ± 13.9	
Tumor dissemination							
Yes	17	4 (2.7–5.3)	34.3 ± 11.8	0.1	7.5 (5.2–9.8)	12.7 ± 8.4	0.13
No	9	7.9 (1.5–14.3)	55.6 ± 16.6		12.1 (0–25.2)	55.6 ± 16.6	
Maximal diameter							
>30 mm	19	4.1 (1.9–6.3)	35.5 ± 11.2	0.124	7.6 (6.2–9)	28.2 ± 10.6	0.455
≤30 mm	12	6.1 (2.4–9.8)	58.3 ± 14.2		8.9 (7.9–9.9)	33.3 ± 13.6	
Bev after progression							
Yes	9	NA	NA	NA	5.13 (4.5–5.8)	NA	0.174; 0.031^e^
No	19	NA	NA		1.2 (0–2.6)	NA	

PFS, progression-free survival; 6m-PFS, progression-free survival rate at 6 months; OS, overall survival; 12m-OS, overall survival rate at 12 months; MGMT, O6-methylguanine DNA-methyltransferase; Bev, bevacizumab; NA, not applicable.

^a^The data were described by median time (95% CI). ^b^The data were described by MEAN ± SEM. ^c^From the log-rank test. ^d^The 95% CI was not reached. ^e^From the Breslow test.

### Toxicity

A total of 139 cycles of chemotherapy was available for safety evaluation. Generally, the toxicities of the combined chemotherapy were relatively well tolerated. No one died from drug-related toxicity. **Table 3** shows all toxicities representing the sum of the highest grade of toxicities attained from every cycle for all patients.

**Table 3:** The toxicities of the combined therapy of apatinib plus temozolomide.

Toxicity^a^	Any Grade (%)	Grades 3 and 4 (%)
**Hematologic**		
Leukopenia	58 (41.7)	5 (3.6)
Neutropenia	41 (29.5)	4 (2.9)
Lymphocytopenia	25 (18.0)	3 (2.2)
Anemia	4 (2.9)	1 (0.7)
Thrombocytopenia	36 (25.9)	6 (4.3)
**Non-hematologic**		
Hypertension	60 (43.2)	8 (5.8)
Hand-foot syndrome	19 (13.7)	1 (0.7)
Proteinuria	19 (13.7)	2 (1.4)
Fatigue	28 (20.1)	0 (0)
Nausea and vomiting	4 (2.9)	0 (0)
Diarrhea	11 (7.9)	1 (0.7)
Constipation	4 (2.9)	0 (0)
Decreased appetite	28 (20.1)	8 (5.8)
Blood bilirubin increased	14 (10.1)	1 (0.7)
Aminotransferase increased	24 (17.3)	2 (1.4)
LDH increased	3 (2.2)	0 (0)
GGT increased	6 (4.3)	1 (0.7)
Hypokalemia	5 (3.6)	0 (0)
Hyponatremia	4 (2.9)	0 (0)
Hypoalbuminemia	3 (2.2)	0 (0)
ST-T change	3 (2.2)	0 (0)
T wave change	9 (6.5)	0 (0)
Right axis deviation	2 (1.4)	1 (0.7)
Erythra	4 (2.9)	0 (0)
Hoarseness	5 (3.6)	0 (0)
Hypothyroidism	8 (5.8)	0 (0)
Gum bleeding	1 (0.7)	0 (0)
Oral ulcer	2 (1.4)	0 (0)

LDH, lactate dehydrogenase; GGT, gamma-glutamyltransferase.

a Ratio of the number of cycles with each toxicity to the total cycles (n = 139).

The most common grade 3 or 4 toxicities were hypertension (5.8%), decreased appetite (5.8%), thrombocytopenia (4.3%), and leukopenia (3.6%). Most of the grade 3 or 4 toxicities were found in the earlier treatment cycles and could be resolved to grade ≤1 (for non-hematologic toxicities) or grade ≤2 (for hematologic toxicities) after symptomatic treatment, dose interruption or reduction and kept grade ≤2 in the follow-up cycles. Hypothyroidism was found in seven of 12 patients with fatigue. Fatigue was alleviated after treatment with levothyroxine sodium and/or apatinib dose reduction.

Dose reduction occurred in nine patients (eight apatinib, one apatinib and temozolomide) due to fatigue (6/9, 66.7%), decreased appetite (3/9, 33.3%) and intolerable palpitations (1/9, 11.1%).

## Discussion

We retrospectively analyzed the efficacy of apatinib plus temozolomide in recurrent glioblastoma and observed a high 6m-PFS rate of 44.6%. The regimen was effective with acceptable toxicities. Moreover, both apatinib and temozolomide are orally administered without the need for hospital admission, meaning that the treatment regimen might have improved adherence and economic effectiveness for patients.

### Effect Comparing With Temozolomide Monotherapy

In this study, the combined regimen could have a superior efficacy to temozolomide monotherapy. Clinical studies demonstrated that the 6m-PFS of the patients with recurrent glioblastoma who received temozolomide on standard 5 days, 7 days on/7 days off or continuous dose-intense schedules was 21, 10 and 23.9%, respectively (17–19). In the DERECTOR trial, 105 patients with glioblastoma at first progression was enrolled and received dose-intensified temozolomide treatment (20). The median PFS and 6m-PFS for 7 days on/7days off regimen were 1.8 months and 17.1%, while that for 3 weeks on/one week off regimen were 2.0 months and 25%.

However, in our study, the median PFS of the whole patients was 4.9 months and the 6m-PFS was 44.6%, which were better that that of temozolomide. Moreover, two patients had long PFS of 30.9 and 38.7+ months, surpassing their prior recurrence intervals of 12 and 14 months, respectively. These suggested that apatinib was likely to enhance the effect of temozolomide, which is consistent with the results of several preclinical studies, which have demonstrated that inhibiting VEGFR-2 could enhance the efficacy of temozolomide. *In vitro*, down-regulated VEGFR-2 results in decreased cell proliferation and higher sensitivity of glioma cells to temozolomide-induced G2 cell cycle arrest (21). In an intracranial murine xenograft model, inhibiting VEGFR-2 suppressed glioblastoma growth and prolonged mouse survival time, both of which were augmented by the incorporation of temozolomide (22).

### Effect Comparing With Bevacizumab-Based Therapy

**Table 4** lists the main study of bevacizumab in recurrent glioblastoma in recent years (4–6, 23, 24). A phase II randomized non-comparative trial of bevacizumab with or without irinotecan in recurrent glioblastoma participants in first or second relapse revealed that the ORR was 28–38% and 6m-PFS was 43–50%. Median OS was 9.2 months in the bevacizumab monotherapy arm and 8.7 months in the combination arm (4). The FDA reviewed and determined that the ORR of bevacizumab was 26% and the 6m-PFS was 36%. In the EORTC 26101 phase 3 trial in patients with first relapsed glioblastoma, the 6m-PFS of the bevacizumab plus lomustine group was 30.2%, the median PFS was 4.2 months and median OS was 9.1 months (6). In our study, the ORR was 26.3%, the median PFS was 4.9 months and 6m-PFS was 44.6%. This indicated that the effect of apatinib plus temozolomide was comparable to that of bevacizumab.

**Table 4:** Overview of bevacizumab study in recurrent glioblastoma in recent years.

Year	Author	Agents	Patients No.	Relapse No.	6m-PFS	OS (months)
2009 (4)	Friedman HS	Bev; Bev + IRI	85 82	First or second	42.6% 50.3%	9.2 8.7
2009 (5)	Kreisl TN	Bev	48	First	29%	7.1
2012 (23)	Desjardins A	Bev + TMZ	32	First or second or third	18.8%	8.5
2014 (24)	Taal W	Bev; Bev + CCNU	50 52	first	16% 42%	8 12
2017 (6)	Wick W	Bev + CCNU	288	First	30.2%	9.1
Current study	Ge JJ	Apatinib + TMZ	31	First or second or third	44.6%	8.2

Bev, bevacizumab; IRI, irinotecan; CCNU, lomustine; TMZ, temozolomide; 6m-PFS, progression-free survival rate at 6 months; OS, overall survival.

Moreover, the small molecule apatinib has some characteristics that differ from bevacizumab. Firstly, apatinib is orally administered and convenient to take while bevacizumab is administered intravenously in a clinic or hospital. Secondly, the short half-life of apatinib (9 h for apatinib *versus* 3 weeks for bevacizumab) allows for quick normalization of wound healing in case of urgent surgical intervention (25). In bevacizumab, however, surgery should not be performed for at least 28 days after the last bevacizumab administration.

### Salvage Treatment After Tumor Progression

Our study observed that, after tumor progression from the combined chemotherapy, bevacizumab usage could help to prolong the survival time (5.1 *versus* 1.2 months). This suggested that the current combined regimen did not reduce the sensitivity of tumor to bevacizumab.

Furthermore, in our study, the median time interval between disease progressions to death was 3.2 months, which is shorter than other reports. A possible explanation for this is that only 15 of 29 patients received anti-tumor therapy after tumor progression, including resection, bevacizumab or other antiangiogenic agents.

### Efficacy Based on Genetic Characterization

MGMT promoter methylation is a prognostic factor. Recurrent glioblastoma with unmethylated MGMT has a poor prognosis (20). After treatment with dose-intensified temozolomide, the PFS was only 1.8 months and the 6m-PFS was 6.9% (20). Even treating with bevacizumab and lomustine, the PFS was 3.02 months and the 6m-PFS was 12.7% (6). In contrast with these reports, we observed a superior PFS of 6.1 months and 6m-PFS of 52.6% for the 19 recurrent glioblastoma with unmethylated MGMT.

IDH mutation status is also associated with prognosis. In our study, 20 patients harbored wild-type IDH, 15 of which were also MGMT unmethylated. For all the 20 patients with wild-type IDH, the median PFS and 6m-PFS were 5.7 months and 48.7%. Wang Y et al. reported that, after treatment of apatinib plus temozolomide, 19 recurrent glioblastoma with wild-type IDH got a PFS of 5.9 months and 6m-PFS of 47.4%, which was similar to our study (26). However, in that study, there was no information about the MGMT methylation status, number of relapse, or tumor dissemination.

## Conclusion

The current study preliminarily shows that the combined therapy of apatinib and temozolomide (1) has a promising efficacy, which could be better than that of temozolomide alone, especially for the patients with unmethylated MGMT and wild-type IDH; (2) does not reduce the sensitivity of tumor to bevacizumab; (3) has manageable toxicities; (4) is orally administered without the need for hospital admission. The major limitations of this study are retrospective design and the relatively small sample size. A randomized controlled trial should be conducted to provide more definitive evidence.

## Data Availability Statement

The raw data supporting the conclusions of this article will be made available by the authors, without undue reservation.

## Ethics Statement

The studies involving human participants were reviewed and approved by the Ethics Committee of Sanbo Brain Hospital Capital Medical University. The patients/participants provided their written informed consent to participate in this study. Written informed consent was obtained from the individual(s) for the publication of any potentially identifiable images or data included in this article.

## Author Contributions

JG: Acquisition of data, analysis and drafting, and revising the article. CL, FX, SQ: Acquisition of data, analysis and interpretation of data. ZG: Acquisition of data. CY: Conception and design. JZ: Conception and design of the work and revision of the article. All authors contributed to the article and approved the submitted version.

## Funding

This work was supported by Scientific Research Common Program of Beijing Municipal Commission of Education (Grant number: KM201710025027) and Neuro-oncology Research Program of Chinese Society of Neuro-oncology (Grant number: CSNO-2014-MSD02).

## Conflict of Interest

The authors declare that the research was conducted in the absence of any commercial or financial relationships that could be construed as a potential conflict of interest.
